# *YTHDF1* gene polymorphisms and neuroblastoma susceptibility in Chinese children: an eight-center case-control study

**DOI:** 10.7150/jca.54496

**Published:** 2021-03-05

**Authors:** Jiabin Liu, Jiwen Cheng, Li Li, Yong Li, Haixia Zhou, Jiao Zhang, Suhong Li, Huimin Xia, Jing He, Zhonghua Yang

**Affiliations:** 1 Department of Pediatric Surgery, Guangzhou Institute of Pediatrics, Guangdong Provincial Key Laboratory of Research in Structural Birth Defect Disease, Guangzhou Women and Children's Medical Center, Guangzhou Medical University, Guangzhou 510623, Guangdong, China.; 2 Department of Pediatric Surgery, the Second Affiliated Hospital of Xi'an Jiaotong University, Xi'an 710004, Shaanxi, China.; 3 Kunming Key Laboratory of Children Infection and Immunity, Yunnan Key Laboratory of Children's Major Disease Research, Yunnan Institute of Pediatrics Research, Yunnan Medical Center for Pediatric Diseases, Kunming Children's Hospital, Kunming 650228, Yunnan, China.; 4 Department of Pediatric Surgery, Hunan Children's Hospital, Changsha 410004, Hunan, China.; 5 Department of Hematology, The Second Affiliated Hospital and Yuying Children's Hospital of Wenzhou Medical University, Wenzhou 325027, Zhejiang, China; 6 Department of Pediatric Surgery, the First Affiliated Hospital of Zhengzhou University, Zhengzhou 450052, Henan, China.; 7 Department of Pathology, Children Hospital and Women Health Center of Shanxi, Taiyuan 030013, Shannxi, China.; 8 Department of Pediatric Surgery, Shengjing Hospital of China Medical University, Shenyang 110004, Liaoning, China.

**Keywords:** *YTHDF1*, polymorphism, neuroblastoma susceptibility, m6A modification.

## Abstract

Neuroblastoma is one of the most common life-threatening extracranial tumors that mainly occurs in children, and its genetic etiology remains largely obscure. RNA m6A modification has been thought to play a key role in cancer progression. *YTHDF1* is the critical downstream gene by which RNA m6A modification exerts its functions. Single nucleotide polymorphisms in the *YTHDF1* gene may affect its expression and biological activity, thereby leading to abnormalities in the regulation of downstream m6A-modified RNA and eventually promoting the initiation and development of tumors. Here, we attempted to evaluate the contributions of two polymorphisms (rs6011668 C>T and rs6090311 A>G) in the *YTHDF1* gene to neuroblastoma susceptibility in 898 cases and 1734 controls that originated in China. Odds ratios (ORs) and 95% confidence intervals (CIs) were calculated in the logistic regression models to evaluate the associations between selected polymorphisms and neuroblastoma risk. Overall, either in a single locus or combination analysis, no significant association with neuroblastoma risk was found for either of the two selected polymorphisms. However, the stratified analysis showed that rs6090311AG/GG genotypes significantly reduced the neuroblastoma risk in males (adjusted OR=0.77, 95% CI=0.62-0.96, *P*=0.018). Moreover, we found that subjects with 2 protective genotypes had a lower tumor risk in males than in those with 0-1 protective genotypes (adjusted OR=0.77, 95% CI=0.62-0.96, *P*=0.018). In summary, our study indicates that *YTHDF1* gene polymorphisms may weakly contribute to neuroblastoma susceptibility. Our findings should be further verified by well-designed studies with larger sample sizes.

## Introduction

Neuroblastoma, one of the most frequent extracranial pediatric tumors, derives from sympathetic neural precursors. It mainly occurs in children younger than 1 year of age, and the average age at diagnosis is approximately 17 months 1. It accounts for approximately 10% of pediatric malignancies and almost 15% of pediatric oncology deaths 2. Currently, neuroblastoma is ranked as the third leading cause of cancer-related pediatric deaths around the world 3. Overall, neuroblastoma can be divided into low-, intermediate- and high-risk subgroups based on clinical phenotypes, pathological features, and prognostic factors 4. The clinical symptoms of neuroblastoma are very extensive because of its highly heterogeneous nature. For low-risk patients, regardless of whether they are treated with minimal chemotherapy, the survival rate is above 95%; for intermediate-risk patients, which comprise 15% of all neuroblastoma patients, the survival rate is still greater than 80% after multimodal therapy. However, nearly half of patients are categorized as high-risk neuroblastoma, and despite intensive comprehensive treatments, the 5-year survival rate remains less than 40% 5, 6. The widespread dissemination and metastasis of cancer cells at the time of diagnosis may partially contribute to such poor prognoses 7. Moreover, survivors have great difficulty marrying and employing due to their lifelong serious coexisting health issues, which impose a great burden on affected families and society 8. For this reason, it is urgent to identify the risk factors for neuroblastoma.

The etiology of neuroblastoma has not yet been fully clarified, and no risk factors have been well documented to influence neuroblastoma susceptibility. Some epidemiological studies have proposed that environmental factors, such as wood dust, radiation sources, and hydrocarbons, may contribute to neuroblastoma susceptibility 9, 10. However, few children develop neuroblastoma when their parents are exposed to these environmental risk factors, and most children do not 11. Growing evidence has shown that genetic factors play key roles in predisposing patients to neuroblastoma 12, 13. For familial neuroblastoma, germline mutations in the *PHOX2B* 14 and* ALK* 15 genes are largely attributed to cancer risk. However, regarding sporadic neuroblastoma, the most common type of neuroblastoma, its etiology remains largely unclear. Benefitting from the rapid development of high-throughput sequencing and bioinformatics technology, genome-wide association studies (GWASs) have become a powerful tool to study the possible genetic mechanisms of complex diseases, such as human malignancy 16. In the past ten years, several GWASs and subsequent replication studies have been conducted. As a result, a series of neuroblastoma susceptibility genes have been identified, including *CASC15* 17, *BARD1* 18,* DUSP12*, *DDX4*, *IL31RA*, *HSD17B12* 19,* LMO1* 20, *LIN28B*, *HACE1* 21,* CPZ* and *MLF1* 22. Furthermore,* NEFL* 23 and *CDKN1B* 24 have also been found to be related to neuroblastoma susceptibility by candidate gene approaches.

RNA m6A modification is methylated at the N6 position of adenosine within messenger RNAs (mRNAs), microRNAs (miRNAs), and long noncoding RNAs (lncRNAs), which are considered the most pervasive, abundant and important chemical modifications in eukaryotic RNAs. RNA m6A modification usually occurs in the 3′ untranslated terminal region (UTR) 25, near the stop codon and translated near the 5′ UTR in an independent manner 26, therefore influencing all aspects of RNA metabolism, including RNA transcription, processing, translation and transportation. RNA m6A modification is a dynamic and reversible process that can be installed by the methyltransferase complex ('writers') and removed by demethylases ('erasers') 27. The m6A sites can be recognized and bound by some RNA binding proteins ('readers'), leading to different destinies of the target RNA 28. For example, it alters gene expression, which may eventually affect the corresponding cell physiological processes and functions. Mounting evidence proposes that m6A modification is related to tumorigenesis, proliferation, differentiation, invasion and metastasis 29-31. *YTHDF1*, a member of the YTH domain family, functions as the 'reader' module for recognizing and binding to m6A-modified RNA 32 and then promotes target mRNA translation and protein synthesis by interacting with initiation factors 33. In bladder cancer, *METTL3* elevates the m6A level of *CDCP1*, enhancing its translation, which is modulated by *YTHDF1*, and the upregulation of *METTL3* and *CDCP1* is correlated with poor prognosis of bladder cancer 34. In endometrial tumors, m6A methylation of the *AKT* negative regulator *PHLPP2* can facilitate *YTHDF1*-mediated translation of the* PHLPP2* gene, inhibiting the AKT signaling pathway and leading to attenuated cell proliferation, migration and invasion 30. In liver cancer, high expression of *METTL3* and *YTHDF1* is associated with worse overall survival, which leads to elevated m6A levels of the *Snail* gene, a pivotal transcription factor for epithelial-to-mesenchymal transition (EMT), and increases *Snail* expression through *YTHDF1*-mediated translation 35. Thus, it is tempting to speculate that functional single nucleotide polymorphisms (SNPs) in *YTHDF1* may influence its expression and binding ability to m6A-modified RNA, which may deregulate downstream target genes, further causing cell dysfunction and eventually tumorigenesis 36. However, no studies regarding the association between *YTHDF1* gene polymorphisms and neuroblastoma susceptibility have been published. Therefore, we conducted this eight-center case-control study to explore the association between SNPs in the *YTHDF1* gene and neuroblastoma risk in Chinese children.

## Materials and Methods

### Study population

In the current case-control study, 898 cases with neuroblastoma and 1734 cancer-free controls were included, and the demographic characteristics of all participants are displayed in **Table S1**. The studied subjects were recruited from eight different regions of China. The criteria of acceptability for the enrolled subjects were described in a previous publication 13. Written informed consent was provided by all participants or their guardians before the study. The study was approved by the Institutional Review Board of each participating hospital.

### SNP selection and genotyping

Two potentially functional polymorphisms (rs6011668 C>T and rs6090311 A>G) in the *YTHDF1* gene were chosen through the dbSNP database (http://www.ncbi.nlm.nih.gov/) and SNPinfo (http://snpinfo.niehs.nih.gov/). The selection criteria were described previously in detail 13, 37. There was no significant LD (R^2^<0.8) among these two selected SNPs (R^2^=0.094 between rs6011668 C>T and rs6090311 A>G), which was calculated in our previous publication 38. Both SNPs were located in the 5' region near the gene, a crucial region for gene expression regulation by numerous transcription factors. Polymorphisms in this region may influence the binding of transcription factors and the gene transcription of *YTHDF1*. Then, the affected *YTHDF1* gene may further influence its downstream genes and ultimately cause a series of abnormalities in downstream biological functions, including cancer susceptibility. For genotyping, we extracted genomic DNA from the peripheral blood of all participants by a TIANamp Blood DNA Kit (TianGen Biotech Co. Ltd., Beijing, China). Then, the purified DNA samples were added to 96-well plates and diluted to 5 ng/μL, and genotyping of all DNA samples for the selected SNPs was performed in 384-well format by standard TaqMan real-time PCR 39-41. Ten percent of the DNA samples were chosen randomly to genotype again to ensure the authenticity of the results. Two sets of genotype concordance rates reached 100%.

### Statistical analysis

The goodness-of-fit χ^2^ test was applied to check whether the selected SNPs deviated from Hardy-Weinberg equilibrium (HWE) in the controls. The comparisons of demographic distributions and allele frequencies between all cases and controls were conducted by the two-sided chi-square test. The odds ratios (ORs) and 95% confidence intervals (CIs) were used to assess associations between the *YTHDF1* polymorphisms and neuroblastoma susceptibility through logistic regression analysis. Furthermore, adjusted ORs and corresponding 95% CIs adjusted for age and sex were calculated by unconditional multivariate logistic regression analysis. Finally, we performed a stratified analysis based on age, sex, tumor origin site, and clinical stage. All statistical analyses were carried out by SAS software (version 9.4 SAS Institute, NC, USA). When the *P*-value was <0.05, the result was considered to be statistically significant.

## Results

### Associations between *YTHDF1* polymorphisms and neuroblastoma risk

This eight-center case-control study contained 898 cases and 1734 controls, of which genotyping was successfully performed in 896 cases and 1733 controls. As shown in **Table 1**, the genotype frequencies of both selected SNPs were consistent with HWE among the control subjects (HWE=0.518 for rs6011668 C>T and HWE=0.285 for rs6090311 A>G). In the single locus analysis, no significant association was found between the selected polymorphisms and neuroblastoma risk, and the same result was found in the combined analysis.

### Stratification analysis

To evaluate whether the selected *YTHDF1* polymorphisms affect neuroblastoma risk among different subgroups, a stratified analysis was carried out according to age, sex, site of tumor origin, and clinical stage (**Table 2**). We failed to find a significant association between the rs6011668 C>T polymorphism and neuroblastoma risk among subgroups. However, we found that subjects with rs6090311AG/GG genotypes had a lower risk of developing neuroblastoma in the male subgroup (adjusted OR=0.77, 95% CI=0.62-0.96, *P*=0.018) than in the reference group. Further combined analysis showed that subjects harboring 2 protective genotypes had a significantly reduced neuroblastoma risk compared with those with 0-1 protective genotypes in the male subgroup (adjusted OR=0.77, 95% CI=0.62-0.96, *P*=0.018).

## Discussion

To explore the correlation between *YTHDF1* gene polymorphisms and neuroblastoma susceptibility, we conducted the present eight-center case-control study in a Chinese population. To the best of our knowledge, this is the first study to evaluate the association between SNPs within the *YTHDF1* gene and neuroblastoma risk. However, neither of the two studied SNPs was correlated with neuroblastoma risk.

*YTHDF1*, which is localized on chromosome 20q11, plays a “reader” role in the m6A modification pathway. As an RNA-binding protein, it functions as a translation regulator by specifically binding to m6A-modified mRNA and then promoting cap-dependent translation 26, enhancing ribosome loading on m6A-containing mRNA and recruiting translation initiation factors, such as eIF3A or eIF3B, to promote the translation efficiency of targeted mRNA 33. Aberrant expression of *YTHDF1* may change the translation efficiency and the expression level of downstream targeted genes, alter the biological functions of cells, and eventually induce oncogenesis. Dysregulation of m6A modification has been shown to be closely related to the initiation and progression of various cancers, such as hepatocellular carcinoma, lung cancer, and acute myeloid leukemia. Numerous studies have shown that *YTHDF1* is overexpressed in various cancers, such as colorectal cancer 42, hepatocellular carcinoma 43, breast cancer 44, Merkel cell carcinoma 45, non-small cell lung cancer 46, and ovarian cancer 47, which are closely associated with an increased risk of these cancers. In colorectal cancer, upregulated *YTHDF1* could stabilize transcripts of the oncogene *C-MYC* and then promote tumor cell proliferation 48. Furthermore, key Wnt signaling components, such as *TCF4*, *DVL3*, and *FZD7*, and the β-catenin major transcriptional effector *TCF7L2* are direct targets of *YTHDF1*, and *YTHDF1* can activate the Wnt/β-catenin signaling pathway by regulating these targeted genes and then promote intestinal stemness and tumorigenesis of colorectal cancer 49. Moreover,* YTHDF1* can directly target *EIF3C* and augment *EIF3C* translation in an m6A-dependent manner, facilitating the tumorigenesis and metastasis of ovarian cancer 47.

Increasing evidence has indicated that genetic variations, such as copy number variation (CNV) and SNPs, which are related to m6A modification modulators, are closely correlated with the malignant progression of various cancers 42, 50, 51. DNA copy number gain is a key cause of aberrant overexpression of oncogenes in cancer 52. There is a certain association between DNA copy number amplification and *YTHDF1* overexpression. Bai et al. found that *YTHDF1* is upregulated in colorectal cancer, and the gain of copy number may be a major mechanism driving the overexpression of *YTHDF1* 42. One study performed by Liu et al. showed that the *YTHDF1* gene was upregulated by frequent amplification in high-grade serous ovarian cancer 47. Importantly, oncogene overexpression driven by gene amplification may be a crucial event during cancer evolution. Furthermore, functional SNPs in gene regulatory regions may also alter gene expression rather than contributing to cancer risk. Numerous studies have reported that SNPs in oncogenes or tumor suppressor genes can modify cancer susceptibility 21-24, 53. However, few studies have been conducted regarding *YTHDF1* gene polymorphisms and cancer risk.

To date, only one other study, performed by Meng et al. 54, has assessed the association between SNPs in the *YTHDF1* gene and cancer susceptibility. However, they failed to find any relationship between SNPs rs2024768 and rs6090289 in the *YTHDF1* gene and colorectal cancer risk, but they revealed that SNP rs118049207 located in the *SND1* gene could modify the mRNA expression of *SND1* and then change the m6A level. Furthermore, the SNP rs118049207 was shown to be associated with colorectal cancer susceptibility. In our recent study, we evaluated the association between two SNPs (rs6011668 C>T and rs6090311 A>G) in the *YTHDF1* gene and hepatoblastoma susceptibility. The results showed that rs6011668 C>T was not associated with hepatoblastoma susceptibility; however, participants with the rs6090311 G allele had a significantly decreased risk of hepatoblastoma. In subsequent expression quantitative trait locus (eQTL) analysis, we found that the rs6090311 G allele was related to reduced expression of the *BIRC7*, *RP5-963E22.4* and *NKAIN4* genes 38.

To the best of our knowledge, this current study is the first to explore the correlation between *YTHDF1* gene polymorphisms and neuroblastoma susceptibility. In this eight-center case-control study, we assessed whether two SNPs (rs6011668 C>T and rs6090311 A>G) affect neuroblastoma susceptibility, which is located in the 5' region of *YTHDF1*, a vital region for regulating gene expression. We failed to find any relationships between the selected SNPs and neuroblastoma risk in the single locus or combination analysis. The paradoxical results may be attributed to different types of cancer. However, in the stratified analysis, we found that the rs6090311AG/GG genotype significantly decreased the neuroblastoma risk in males, and the participants with 2 protective genotypes had a reduced tumor risk in males when compared to those with 0-1 protective genotypes. It should be noted that these positive results may have been due to the relatively small sample size in the stratification analysis.

Several limitations of this study should be mentioned. First, the sample size remained relatively moderate even though the subjects in this study were recruited from eight independent hospitals, especially for the stratification analysis. Second, only two SNPs in the *YTHDF1* gene were evaluated, and more potentially functional SNPs in the *YTHDF1* gene should be investigated. Third, the participants involved in this study were of Chinese origin; therefore, the conclusions obtained from this study may not be suitable for other ethnicities. Fourth, only genetic analysis was conducted on neuroblastoma risk, incorporating analysis on environmental factors and genetic-environmental factors. Neuroblastoma is a heterogeneous disease with complicated etiologies.

In conclusion, our present results indicate that *YTHDF1* polymorphisms (rs6090311 A>G) may affect neuroblastoma susceptibility in a low-penetrance and sex-dependent manner. Well-designed studies with a larger sample size are needed to verify our conclusion. Furthermore, mechanistic research should be carried out to expound on the underlying mechanisms by which *YTHDF1* gene polymorphisms affect neuroblastoma susceptibility.

## Supplementary Material

Supplementary table.

## Figures and Tables

**Table 1:** Association between *YTHDF1* gene polymorphisms and neuroblastoma susceptibility

Genotype	Cases (N=896)	Controls (N=1733)	*P*^ a^	Crude OR (95% CI)	*P*	Adjusted OR (95% CI) ^b^	*P*^ b^
rs6011668 C>T (HWE=0.518)							
CC	647 (72.21)	1258 (72.59)		1.00		1.00	
CT	229 (25.56)	441 (25.45)		1.01 (0.84-1.22)	0.919	1.01 (0.84-1.22)	0.885
TT	20 (2.23)	34 (1.96)		1.14 (0.65-2.00)	0.639	1.16 (0.66-2.04)	0.597
Additive			0.751	1.03 (0.87-1.21)	0.751	1.03 (0.88-1.21)	0.703
Dominant	249 (27.79)	475 (27.41)	0.836	1.02 (0.85-1.22)	0.836	1.02 (0.86-1.23)	0.793
Recessive	876 (97.77)	1699 (98.04)	0.643	1.14 (0.65-1.99)	0.644	1.16 (0.66-2.03)	0.604
rs6090311 A>G (HWE=0.285)							
AA	374 (41.74)	667 (38.49)		1.00		1.00	
AG	411 (45.87)	833 (48.07)		0.88 (0.74-1.05)	0.148	0.88 (0.74-1.04)	0.137
GG	111 (12.39)	233 (13.44)		0.85 (0.66-1.10)	0.218	0.84 (0.65-1.09)	0.189
Additive			0.121	0.91 (0.81-1.03)	0.121	0.91 (0.80-1.02)	0.103
Dominant	522 (58.26)	1066 (61.51)	0.106	0.87 (0.74-1.03)	0.106	0.87 (0.74-1.02)	0.094
Recessive	785 (87.61)	1500 (86.56)	0.446	0.91 (0.72-1.16)	0.447	0.90 (0.71-1.15)	0.405
Combined effect of protective genotypes ^c^							
0	20 (2.23)	34 (1.96)		1.00		1.00	
1	354 (39.51)	633 (36.53)		0.95 (0.54-1.68)	0.861	0.94 (0.53-1.66)	0.825
2	522 (58.26)	1066 (61.51)		0.83 (0.47-1.46)	0.523	0.82 (0.47-1.44)	0.483
0-1	374 (41.74)	667 (38.49)		1.00		1.00	
2	522 (58.26)	1066 (61.51)	0.106	0.87 (0.74-1.03)	0.106	0.87 (0.74-1.02)	0.094

^a^ χ^2^ test for genotype distributions between neuroblastoma cases and controls. ^b^ Adjusted for age and sex. ^c^ Protective genotypes were rs6011668 CC/CT and rs6090311 AG/GG.

**Table 2:** Stratification analysis for the association between *YTHDF1* genotypes and neuroblastoma susceptibility

Variables	rs6011668 (case/control)		Adjusted OR ^a^	*P* ^a^	rs6090311 (case/control)		Adjusted OR ^a^	*P* ^a^	Protective genotypes (case/control)		Adjusted OR ^a^	*P* ^a^
	CC	CT/TT	(95% CI)		AA	AG/GG	(95% CI)		0-1	2	(95% CI)	
Age, month												
≤18	237/517	107/196	1.19 (0.90-1.58)	0.228	155/276	189/437	0.77 (0.59-1.00)	0.051	155/276	189/437	0.77 (0.59-1.00)	0.051
>18	410/741	142/279	0.91 (0.72-1.16)	0.457	219/391	333/629	0.95 (0.76-1.17)	0.599	219/391	333/629	0.95 (0.76-1.17)	0.599
Sex												
Female	298/531	108/213	0.91 (0.69-1.19)	0.481	158/294	248/450	1.01 (0.79-1.30)	0.915	158/294	248/450	1.01 (0.79-1.30)	0.915
Male	349/727	141/262	1.13 (0.89-1.44)	0.333	216/373	274/616	**0.77 (0.62-0.96)**	**0.018**	216/373	274/616	**0.77 (0.62-0.96)**	**0.018**
Sites of origin												
Adrenal gland	184/1258	64/475	0.92 (0.68-1.25)	0.606	106/667	142/1066	0.83 (0.63-1.09)	0.172	106/667	142/1066	0.83 (0.63-1.09)	0.172
Retroperitoneal	231/1258	87/475	1.01 (0.77-1.32)	0.949	129/667	189/1066	0.91 (0.71-1.16)	0.437	129/667	189/1066	0.91 (0.71-1.16)	0.437
Mediastinum	151/1258	62/475	1.08 (0.79-1.48)	0.635	93/667	120/1066	0.81 (0.61-1.08)	0.153	93/667	120/1066	0.81 (0.61-1.08)	0.153
Others	70/1258	35/475	1.31 (0.86-2.00)	0.204	42/667	63/1066	0.95 (0.63-1.42)	0.790	42/667	63/1066	0.95 (0.63-1.42)	0.790
INSS stage												
I+II+4s	341/1258	128/475	0.99 (0.79-1.24)	0.926	203/667	266/1066	0.82 (0.67-1.01)	0.064	203/667	266/1066	0.82 (0.67-1.01)	0.064
III+IV	284/1258	110/475	1.04 (0.81-1.33)	0.768	155/667	239/1066	0.95 (0.75-1.19)	0.627	155/667	239/1066	0.95 (0.75-1.19)	0.627

^a^ Adjusted for age and sex, omitting the corresponding stratification factor.
