# Supplementary material for: YTHDF1 gene polymorphisms and neuroblastoma susceptibility in Chinese children: an eight-center case-control study
Source: J Cancer. 2021 Mar 5;12(8):2465–71. doi: 10.7150/jca.54496 (PMC7974895; doi:10.7150/jca.54496)
Supplement: Supplementary file 1 — Supplementary table. [file jcav12p2465s1.pdf]

Table S1. Frequency distribution of selected characteristics in neuroblastoma cases and cancer-free controls from eight hospitals

Variables	Combined subjects (8 Centers)				
	Cases (n=898)		Controls (n=1734)		*P* [a]
	No.	%	No.	%	
Age range, month	0.00-176.00		0.004-156.00		0.155
Mean ± SD	33.11±28.07		30.41±24.90		
≤18	344	38.31	714	41.18	
>18	554	61.69	1020	58.82	
Sex					0.236
Female	407	45.32	744	42.91	
Male	491	54.68	990	57.09	
INSS stages					
I	310	34.52	/	/	
II	160	17.82	/	/	
III	163	18.15	/	/	
IV	231	25.72	/	/	
4s	18	2.00	/	/	
NA	16	1.78	/	/	
Sites of origin					
Adrenal gland	248	27.62	/	/	
Retroperitoneal region	319	35.52	/	/	
Mediastinum	214	23.83	/	/	
Other region	105	11.69	/	/	
NA	12	1.34	/	/	

SD, standard deviation, NA, not available.

[a] Two-sided χ^2 test for distributions between neuroblastoma cases and cancer-free controls.
